# Ethanol and High-Value Terpene Co-Production from Lignocellulosic Biomass of *Cymbopogon flexuosus* and *Cymbopogon martinii*

**DOI:** 10.1371/journal.pone.0139195

**Published:** 2015-10-05

**Authors:** Blake L. Joyce, Valtcho D. Zheljazkov, Robert Sykes, Charles L. Cantrell, Choo Hamilton, David G. J. Mann, Miguel Rodriguez, Jonathan R. Mielenz, Tess Astatkie, C. Neal Stewart

**Affiliations:** 1 Department of Plant Sciences, University of Tennessee, Knoxville, TN, 37996, United States of America; 2 Columbia Basin Agricultural Research Station, Oregon State University, 48037 Co 788 Rd, Adams, OR, 97810, United States of America; 3 National Renewable Energy Laboratory, Golden, CO, 80401, United States of America; 4 BioEnergy Science Center, Oak Ridge National Laboratory, Oak Ridge, TN, 37831, United States of America; 5 Agricultural Research Service, United States Department of Agriculture, NPURU, University, MS, 38677, United States of America; 6 Oak Ridge National Laboratory, Environmental Science Division, Oak Ridge, TN, 37831, United States of America; 7 Faculty of Agriculture, Dalhousie University, 50 Pictou Road, P.O. Box 550, Truro, NS, B2N 5E3, Canada; National Renewable Energy Lab, UNITED STATES

## Abstract

*Cymbopogon flexuosus*, lemongrass, and *C*. *martinii*, palmarosa, are perennial grasses grown to produce essential oils for the fragrance industry. The objectives of this study were (1) to evaluate biomass and oil yields as a function of nitrogen and sulfur fertilization, and (2) to characterize their utility for lignocellulosic ethanol compared to *Panicum virgatum* (switchgrass). Mean biomass yields were 12.83 Mg lemongrass ha^-1^ and 15.11 Mg palmarosa ha^-1^ during the second harvest year resulting in theoretical biofuel yields of 2541 and 2569 L ethanol ha^-1^ respectively compared to reported 1749–3691 L ethanol ha^-1^ for switchgrass. Pretreated lemongrass yielded 198 mL ethanol (g biomass)^-1^ and pretreated palmarosa yielded 170 mL ethanol (g biomass)^-1^. Additionally, lemongrass yielded 85.7 kg essential oil ha^-1^ and palmarosa yielded 67.0 kg ha^-1^ with an estimated value of USD $857 and $1005 ha^-1^. These data suggest that dual-use crops such as lemongrass and palmarosa may increase the economic viability of lignocellulosic biofuels.

## Introduction

Replacing petroleum as a natural resource extends beyond producing renewable liquid fuels. Petroleum products shape modern life: plastics, lubricants, asphalt, and petrochemicals for reagents in chemical synthesis, synthetic fibers for textiles, cosmetics, flavoring and food additives, surfactants and cleaning chemicals. The petroleum industry currently benefits from over 100 years of optimization and infrastructure development whereas modern biobased products, i.e. liquid biofuels and renewable coproducts, are comparatively a nascent industry. As such, the developing bioproducts industry can benefit from utilizing petroleum economic strategies such as production of high-value commodities alongside low-value fuel chemicals and take advantage of established infrastructure and transportation. These strategies can be employed in tandem with designing optimized fermentation or thermochemical conversion processes suitable for the unique nature of renewable bioproducts.

Liquid fuels are currently necessary to maintain modern transportation and industrial infrastructure. However, the feasibility of producing renewable biofuels is inherently linked to the economic viability of production. In the United States, roughly 90% of crude oil is converted for use as liquid fuels; however, the 7–8% of crude oil that produces high-value chemical commodities accounts for an estimated 25–35% of annual profits [1]. Coproduction of high-value commodities from lignocellulosic sources has recently become a major focus of research for these reasons. However, examples of coproduction of high-value commodities with biofuels from lignocellulosic feedstocks and broad-scale economic evaluation of these examples are still lacking.

Lemongrass [*Cymbopogon flexuosus* (Steud.) Wats, (syn. *Andropogon nardus* var. *flexuosus* Hack; *A*. *flexuosus* Nees)] and palmarosa [*Cymbopogon martini* (Roxb.) Wats. var *martinii* (syn. C. *martini* Sapg var. *motia*)] are subtropical essential oil plants [2]. Both crops are produced on large tracts of land on multiple continents. The countries with significant production are: Guatemala, Brazil, China, India, Indonesia, Haiti, Madagascar, and other Eastern African countries. China and Indonesia are the major producers of lemongrass oil accounting for 40% of the world production, which is estimated to be between 800 and 1300 tons of oil year^-1^. India and Brazil produce the majority of palmarosa oil, which is estimated at around 100 tons of oil year^-1^ for each country [2].

Terpenoid hydrocarbons (major constituents of lemongrass and palmarosa oils) have been investigated as potential, drop-in ready advanced biofuel chemicals for more than three decades [3–5]. Therefore, terpenoids have both economic advantages, i.e. a diversity of markets and high-value, in addition to advantages as biofuels, i.e. fungibility with existing liquid fuels and positive low temperature operability properties. Although terpenoids are interesting for these beneficial economic and fuel advantages, they also typically have antimicrobial activities that may inhibit microbial fermentation in biorefineries. Both citral and geraniol have been shown to have antimicrobial activities against *Saccharomyces cerevisiae* [6,7].

This study seeks to explore the use of dual or multi-use biomass. Specific objectives of this study were (1) to evaluate *C*. *flexuosus* and *C*. *martinii* biomass yields, oil yields and composition as a function of N and S fertilization to determine yields of oleoresins and biomass for biofuel and high-value coproduct applications, (2) to characterize the cell wall components of the biomass, and its usefulness for fermentation to produce lignocellulosic ethanol from *Saccharomyces cerevisiae*, and (3) to evaluate this system for potential implications in direct-production of advanced biofuels and high-value coproducts with plant biomass.

## Materials and Methods

### Field experiments

In 2008 and in 2009 cropping seasons, a field experiment was carried out at the Mississippi State University North MS Research and Extension Center (NMREC) in Verona, Mississippi, USA (34°43’22” N and -88°43’22” W). Dr. Alan Blaine, the Director of the NMREC provided permission for the use of the land at the Center. The field studies did not involve any endangered or protected species. Certified *C*. *flexuosus* and *C*. *martinii* were purchased from Richters (ON, Canada). Transplants were produced in a double-plastic controlled temperature greenhouse during March-April. Lemon grass and palmarosa seeds were sown in Metromix 300 (The Scotts Co., Marysville, OH) growth medium, in 48-cell plastic trays to provide one transplant per cell. The production of transplants in the greenhouse continued for 45 days. The temperature was maintained at 22 to 25°C during the day and 18°C at night. Nutrients were provided with weekly fertilization with 1.8 g of 20-20-20 N-P_2_O_5_-K_2_O in 300 mL of water. In addition, transplants were top irrigated daily. Lemongrass and palmarosa seedlings (approximately 12 and 15 cm, respectively) were transplanted into the field in May 2008 and again in May 2009. Plot size was 1.4 x 6 m, and 12 lemongrass and 12 palmarosa plants were transplanted in each plot, into two rows, at 60 cm in-row spacing on each bed. Beds were spaced at 180 cm apart.

The soil at the experimental site was composed of Quitman sandy loam (fine-loamy, siliceous, semiactive, thermic, Aquic Paleudult). Glyphosate at 2 kg ha^-1^ was applied prior to land preparation, which included disking two weeks after the herbicide application. Prior to land preparation, soil samples (0–15 cm deep, 3 composite samples made of 24 soil cores) were analyzed for extractable nutrients. Before transplanting, phosphorus (P) and potassium (K) fertilizers were applied to ameliorate deficiencies based on soil test reports. Lemongrass and palmarosa plants were planted in previously prepared raised beds (12 cm high and 77 cm wide across the top). The raised beds were prepared by using a press-pan-type bed shaper machine. The machine also placed plastic mulch on the top of the bed and a drip tape irrigation tube at 2–3 cm soil depth below the soil surface, in the middle of the bed.

The N fertilizers (as ammonium nitrate) and S fertilizer (as sulfur bentonite, 90% S) were applied in the middle of the bed, depending on the treatments, and approximately 2 weeks after transplanting. Aboveground portions of lemongrass and palmarosa plants were harvested using a hedge trimmer at approximately 20 cm above the soil surface. Two harvests were taken from each of the crops every year. In 2008, lemongrass harvest 1 was on September 23^rd^, and harvest 2 was on October 28^th^, while palmarosa harvest 1 was on September 24^th^ and harvest 2 was on October 27^th^. In 2009, lemongrass harvest 1 was on September 28^th^ and harvest 2 was on October 20^th^, while palmarosa harvest 1 was on September 28^th^, and harvest 2 was on October 26^th^. Whole, above ground plant parts were weighed, air dried in a shaded greenhouse (at approximately 40°C), reweighed to record dry weight, and plants were steam distilled for extraction of essential oil.

BioEnergy Science Center (BESC) *Panicum virgatum* ‘Alamo’ standard was used as an external control. A lot was obtained from the National Renewable Energy Laboratory, Golden, CO. *P*. *virgatum* ‘Alamo’ seed was purchased from MBS Seeds LTD (Denton, TX) which was produced in 2006 (Lot #6011A) with an effective germination rate of 30%. The standard was grown at the Samuel Roberts Noble Foundation in Ardmore, Oklahoma and planted on June 11^th^, 2007 with 17.8 cm row spacing and a seeding rate of 30%. The stand was harvested on November 2^nd^, 2007 and baled on November 5^th^, 2007. The 0.2 ha stand was fertilized with 99.8 kg of 46-0-0 NPK fertilizer yr^-1^ for a rate 45.9 kg N 0.2 ha^-1^ (229.6 kg N ha^-1^).

### Essential oil extraction

Essential oil was extracted via steam distillation (300 g from lemon grass and 250 g from palmarosa) in a 2 L steam distillation unit for 60 min as described previously [8,9]. The different sample sizes were due to unequal amount of biomass from the two crops that can fit the same 2 L bioflask. Immediately after the end of the distillation, the essential oil was separated, measured on an analytical scale and stored at minus 5°C for further analysis. The essential oil content of lemongrass and palmarosa was calculated as the weight of oil in g g^-1^ dry plant tissue.

### Gas Chromatography—Mass Spectroscopy Quantitative Analysis

Quantitative analyses of oil samples were performed using gas chromatography—mass spectroscopy (GC-MS) at the National Center for Natural Products Research in Oxford, MS, using the GC-MS methods described in [10].

Commercial standards (*R*)-(+)-limonene (CAS 95327-98-3) and (+)-δ-cadinene (CAS 483-76-1) were purchased from Fluka (Switzerland); citral (geranial and (*Z*)-citral; CAS 5392-40-5), geraniol (CAS 106-24-1), geranyl acetate (CAS 105-87-3), (-)-*trans*-caryophyllene (CAS 87-44-5), and caryophyllene oxide (CAS 1139-30-6) were purchased from Sigma-Aldrich (St. Louis, MO, USA). With five concentration points, an external standard least squares regression for quantification was used. Each specific analyte was used to formulate a separate calibration curve using MS total ion chromatogram (TIC) data. Linearity was imposed by using response factors and regression coefficients independently. Response factors were calculated using the equation RF = DR/*C*, where DR was the detector response in peak area (PA) and *C* was the analyte concentration. Since citral was only available as a mixture of *E* (geranial) and *Z* ((Z)-citral) isomers, the TIC area from both isomers was added together to generate the response factor used for the two individual isomers which were quantified separately using that same RF.

The chromatograms of each of the essential oil samples from the field experiments were compared to the chromatograms from standards. Target analytes were confirmed by retention time and mass spectra. Confirmed integrated peaks were used to determine percentage of each chemical constituent in the essential oil itself. The RF of the target chemical constituent was used to determine the percentage of oil for each sample using the equation PA/RF/*C* x 100 = % analyte in the oil on a wt/wt basis.

### Statistical analysis

Repeated measures analysis of the data collected from the experiments conducted at Verona, Mississippi in 2008 and 2009 were analyzed together with six blocks, composed of the combinations of the two years and the three blocks in the field. The two harvests were used as the time points for the repeated measures analysis completed using the Mixed Procedure of SAS [11]. The analysis was completed using two models; one with both crops in which comparison of the crops is made in terms of the responses, and one for each crop where such and between-crop comparison is not made. We, therefore, had three separate analyses: 1) for dry weight yield and essential oil yield responses, a 2×4×4 factorial in six blocks, with the factors of interest being crop (lemongrass [LG], palmarosa [P]), N (0, 40, 80, 160 kg ha^-1^), and S (0, 30, 60, 90 kg ha^-1^) and the repeated measures factor, harvest (1, 2) was used; 2) for essential oil (EO) content, and the composition and yield of β-caryophyllene, (*Z*)-citral and (E)-citral of lemongrass, a 4×4 factorial in six blocks; and 3) for essential oil content, and the composition and yield of geraniol and geranylacetate of palmarosa, a 4×4 factorial in six blocks were used. For both 2) and 3) models, the factors of interest were N (0, 40, 80, 160) and S (0, 30, 60, 90) applications, and Harvest (1, 2) as the repeated measures factor. For each response, the validity of model assumptions was verified by examining the residuals as described in [12]. Some of the responses required cubic root transformation to achieve normality of the error terms, however, the means shown in the tables and figures are back-transformed to the original scale. For significant (p-value < 0.05) and marginally significant (p-value between 0.05 and 0.1) effects, further multiple means comparison was completed for by comparing the least squares means of the corresponding treatment combinations. Letter groupings were generated using a 1% level of significance for two-factor and three-factor interaction effects, and using a 5% level of significance for main effects.

### Pretreatment, ethanol fermentation, and high performance liquid chromatography analysis

Dried *C*. *martinii* and *C*. *flexuosus* biomass samples from field trials were ground in a Wiley mill with a 1 mm screen. A portion of whole dried plant biomass was separated into leaf and stem biomass for biomass composition analysis. Bench-scale ethanol fermentations and pretreatment of biomass were conducted as described previously [13]. Unless stated otherwise, 3.0 g (15% of total fermentation volume) of dried biomass was loaded into each 70 mL Septi-Chek glass vials (Becton Dickson, http://www.bd.com), and fermentations were done in triplicate. The fermentation microbe was *S*. *cerevisiae* D5α culture and enzyme was Accellerase 1500 (Genencor, Rochester, NY). The solution was diluted to 20 mL total volume, weighed for calculation of weight loss in later time points, and placed in a 37°C incubator. At each time point, the bottles were removed from the incubator, and the cap of the bottle was pierced with a needle to allow carbon dioxide to escape from the closed vessel. The remaining weight was then recorded and weight loss was calculated.

After the fermentations were complete, the remaining biomass solutions were centrifuged and 1.0 mL of the supernatant was removed with a 1 mL syringe and filtered through a 13 mm syringe 0.2 μm filter (Millipore, Massachusetts). Ethanol and fermentation liquids were quantified by HPLC (Agilent 1200 Series LC system with 1200 Series refractive index detector) equipped with an Aminex HPX-87P column (Agilent Technologies).

### Determination of saccharification efficiency and lignin composition

Enzymatic saccharification efficiency of all biomass samples were carried out using a high-throughput plate hydrothermal pretreatment and enzymatic saccharification procedure developed at the National Renewable Energy Laboratory [14]. Samples were run twice with three technical replicates for each run. Lignin composition, content, and S/G ratio was determined using pyrolysis molecular beam mass spectrometry (PyMBMS) method as previously described [15]. Samples were run with three technical replicates for each plant for enzymatic saccharification and with two technical replicates for PyMBMS.

## Results and Discussion

Currently, lignocellulosic biofuel production models have a similar logical flow despite the many different technological systems that are being investigated. First, plant feedstocks are grown, collected, processed, and transported to biorefinery facilities. Second, plant feedstocks are converted into fuels and potential coproducts either through biological methods, e.g. saccharification and fermentation, or through chemical methods, e.g. thermochemical conversion. Last, products from biomass are converted into final market products and unused biomass fractions are processed as waste and most commonly burned to generate heat [16]. To date, plant biofuel feedstock research has focused primarily on reduction of lignocellulosic feedstock recalcitrance and increasing overall biomass yield. Likewise, investigation into mechanisms to produce chemicals of interest, i.e. biofuels and coproducts, has occurred almost exclusively at the microbial and thermochemical conversion steps. However, this logical flow ignores the potential for plant feedstocks to produce myriad biochemicals that can be used as biofuels or coproducts directly from sunlight.

We sought here to examine the capability of relatively novel feedstocks to yield biofuel and established high-value coproducts at a pilot-scale system in tandem. *Cymbopogon flexuosus* and *C*. *martinii* offer a unique opportunity for agronomic production of biofuel and coproducts. Though *C*. *flexuosus* and *C*. *martinii* are native to Southeast Asia, we selected *C*. *flexuosus* and *C*. *martinii* specifically as an agronomically-relevant model system as their agricultural production mirrors switchgrass, they naturally produce high-value coproducts, and the high-value coproducts are composed of terpenoids that have been proposed for use as advanced biofuels. Additionally, the terpenoids in *C*. *flexuosus* and *C*. *martinii* essential oils have been directly linked to *S*. *cerevisiae* inhibition and toxicity [6,7]. Taken together, these species offer two attractive model systems to study *in planta* production of advanced biofuels and coproducts.

However, several considerations need to be investigated to understand the feasibility of direct-coproduct biosynthesis in lignocellulosic feedstock crops whether through breeding or biotechnological modification: 1) agronomic inputs and resulting yields that offset costs of inputs, 2) biochemical coproduct inhibition of downstream fermentation processing for biofuel production, and 3) economically feasible ways to remove plant-derived coproducts. Here we investigate the first two considerations for a dual-use lignocellulosic crops, i.e. lemongrass and palmarosa, for production of biofuels and low-volume high-value essential oil coproduct.

### C. flexuosus and C. martinii biomass and essential oil yield in field trials

**Fig 1:**
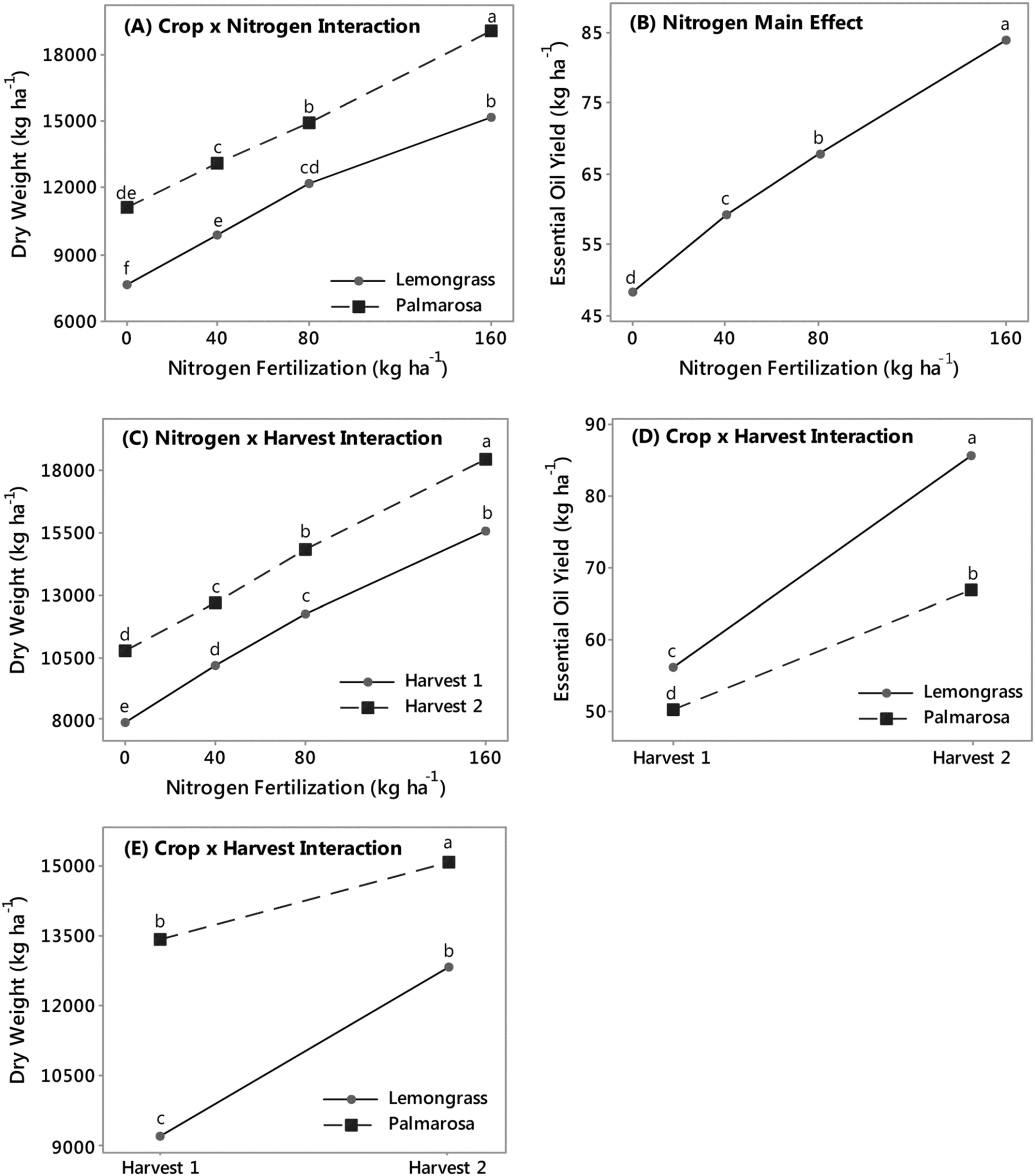
Main and interaction effect plots for dry weight biomass (kg ha^-1^) [(A) Crop x Nitrogen Interaction, (C) Nitrogen x Harvest Interaction, and (E) Crop x Harvest Interaction], and essential oil (kg ha^-1^) [(B) Nitrogen Main Effect, and (D) Crop x Harvest Interaction]. For each plot means sharing the same letter are not significantly different.

Small plot field trials were conducted to determine growing conditions of *C*. *flexuosus* and *C*. *martinii* in the growing regions of the United States. The interaction effect of crop and harvest was significant on dry weight yields and essential oil yield, whereas, the interaction effects of crop and N, and N and harvest were significant on dry weight yield, but not on essential oil yield (S1 Table). Overall, increasing N application rates increased dry weight yields of both lemongrass (ranged from 7 673 to 15 196 kg ha^-1^) and palmarosa (ranged from 11 078 to 19 006 kg ha^-1^), with palmarosa producing more biomass than lemongrass within each application rate of N (Fig 1A). The main effect of N was also significant on essential oil yield (S1 Table). Increasing N application rate brought a stepwise increase in essential oil yields of both crops (Fig 1B). The interaction of N application rate and harvest increased dry weight yields of both crops (Fig 1C). It has been reported that increasing N application rates caused a linear increase in yields of lemongrass biomass up to 150 kg N ha^-1^ [17,18]. Also, [19] reported that under irrigated conditions, 100 kg N ha^-1^ would provide optimal lemongrass yields, while 75 kg N ha^-1^ to 80 kg N ha^-1^ would be sufficient under non-irrigated conditions. Moreover, [9] tested 4 N rates and found 80 kg N ha^-1^ to be sufficient for optimal biomass yields of lemongrass under conditions in Mississippi. Also, [20] concluded 100 kg N ha^-1^ for the establishment year and 150 kg N ha^-1^ for the second growing year optimized palmarosa biomass yield. Contrary to these reports, the current work suggests that both lemongrass and palmarosa biomass yields increase with increasing N fertilization. Interestingly, [21] suggested that optimum biomass production for lowland switchgrass would require approximate N fertilization rates of 100 kg N ha^-1^. Thus, lemongrass and palmarosa will respond similarly to switchgrass under the same management practices.

Within a crop and N application rate, harvest 2 provided higher yields than harvest 1 (Fig 1E). Dry biomass yields from the second year of lemongrass and palmarosa field trials had a mean yield of 12.83 Mg ha^-1^ and 15.11 Mg ha^-1^, respectively (Fig 1C). Upland switchgrass cultivars have been reported to have a mean yield (8.7 ±4.2) Mg ha^-1^ whereas lowland switchgrass cultivars yield (12.9 ±5.9) Mg ha^-1^ across growing ranges, harvest years, land quality, stand size, precipitation, and other agronomic variables [21]. Biomass yield of lemongrass and palmarosa in Mississippi is therefore comparable to switchgrass production ranging from the mean of upland varieties to exceeding upland switchgrass cultivar means depending on nitrogen fertilization rate, crop, and harvest year. However, the lemongrass and palmarosa plots were irrigated in this trial, whereas most switchgrass plots are rainfed which likely had a positive effect on the lemongrass and palmarosa biomass yields.

Generally, essential oil yields of lemongrass were greater than the oil yields of palmarosa within each harvest: 56.1 kg ha^-1^ for lemongrass essential oil and 50.4 kg ha^-1^ for palmarosa from harvest 1, and 85.7 and 67.0 kg ha^-1^, respectively, from harvest 2 (Fig 1D). In general, palmarosa and lemongrass essential oil content (% biomass) and essential oil yields (kg ha^-1^) in Mississippi were similar to those reported for traditional growing countries such as southern India [17,22]. Palmarosa yields were also comparable to yields obtained from 4 harvests and eight N applications [20,23], and palmarosa yields from 6 harvest [24]. Hence, lemongrass and palmarosa in the Southeastern US can provide similar productivity to traditional producing regions in the world. Lemongrass and palmarosa frost hardiness will dictate their growing range in the US. In a previous study, lemongrass had a 30% winter survival after the first year of transplanting into the field in plant hardiness zone 8b, but only 1% in zone 7b [9]. Therefore, the perennial growing range of these crops will be from southern Texas to South Carolina.

### *C*. *flexuosus* essential oil responses

**Fig 2:**
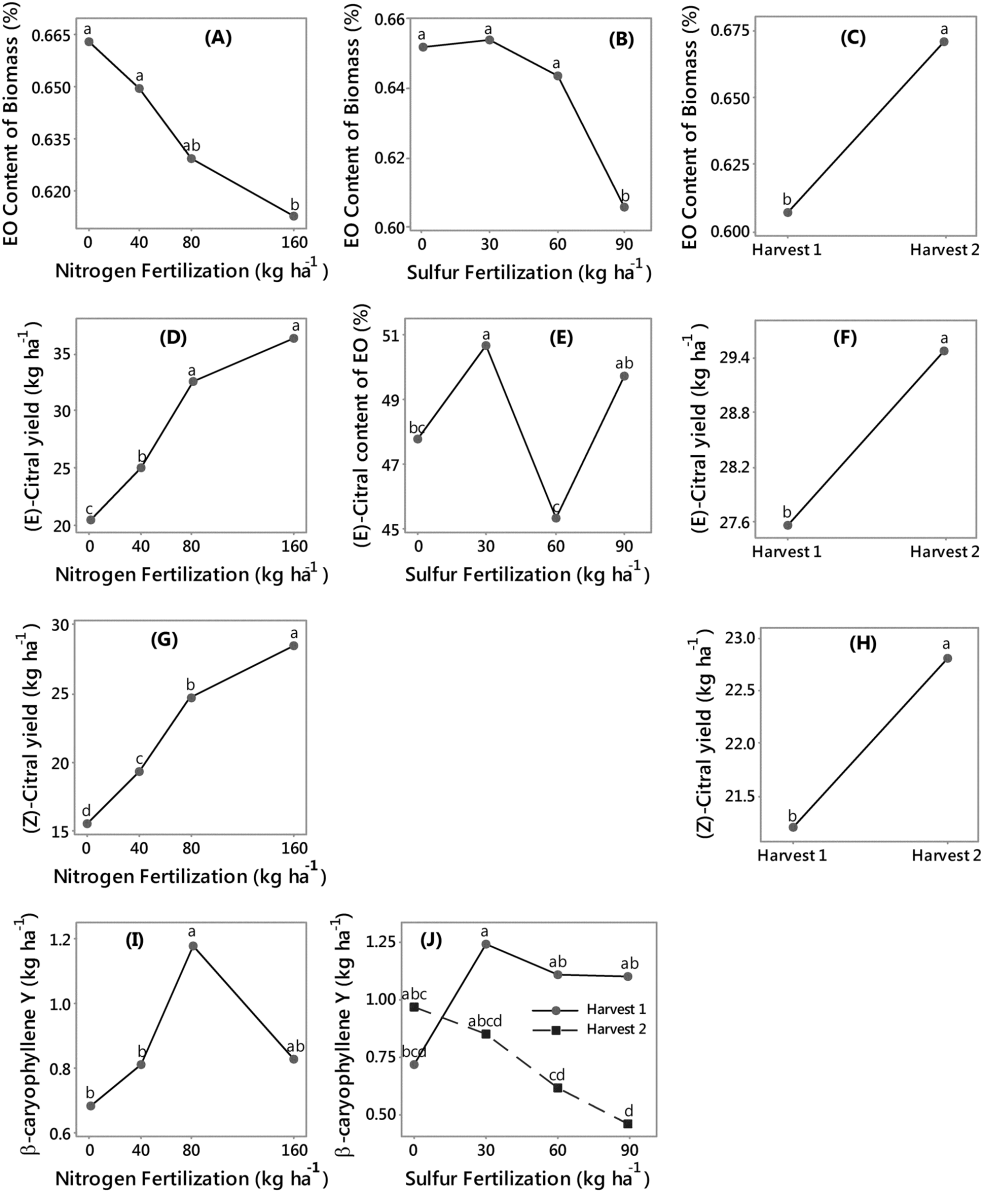
Means of lemongrass essential oil content (%) from the different levels of (A) Nitrogen, (B) Sulfur, and (C) Harvest, (*E*)-citral content (%) from the different levels of (E) sulfur, (*E*)-citral yield (kg ha^-1^) from the different levels of (D) Nitrogen, and (F) Harvest, (*Z*)-citral yield (kg ha^-1^) from the different levels of (G) Nitrogen, and (H) Harvest, and β-caryophyllene yield (kg ha^-1^) from the different levels of (I) Nitrogen, and (J) combinations of Sulfur and Harvest. For each plot, means sharing the same letter are not significantly different.

Additionally, the essential oil characteristics of lemongrass and palmarosa grown on the small field plots were investigated to determine potential yield of high-value secondary coproducts and their response to agronomic parameters such as fertilization rates. The essential oil content of lemongrass was higher in the N0 and N40 treatments (0.66 and 0.65%, respectively), and lower in the N160 treatment (0.61%), indicating that increasing N rates may reduce essential oil content (Fig 2A). The essential oil content was significantly higher in S0, S30 and S60 rates than in the S90 rate (Fig 2B). The essential oil content from harvest 2 was higher than that from harvest 1, 0.67 and 0.61%, respectively (Fig 2C). Previously, lemongrass essential oil content was reported to vary between 0.55 and 1.03% [25] with some selected clones may reach essential oil up to 1.3–1.5% [26]. However, such high-essential oil content clones did not seem to get established as most reports had essential oil content similar to this study. For example, lemongrass essential oil content was found to vary between 0.35 to 0.6% of the dried biomass [9]. N, S, and harvest, separately had a significant effect on lemongrass essential oil; but they all interacted on the concentrations of β-caryophyllene and (*Z*)-citral (S2 Table). The main effect of S was significant, and the interaction effect of N and harvest was marginally significant on the concentration of (*E*)-citral; the main effect of N, and the interaction effect of S and harvest were significant on β-caryophyllene yield; whereas, N and harvest were individually significant on the yields of (*Z*)-citral and geraniol (S2 Table).

**Fig 3:**
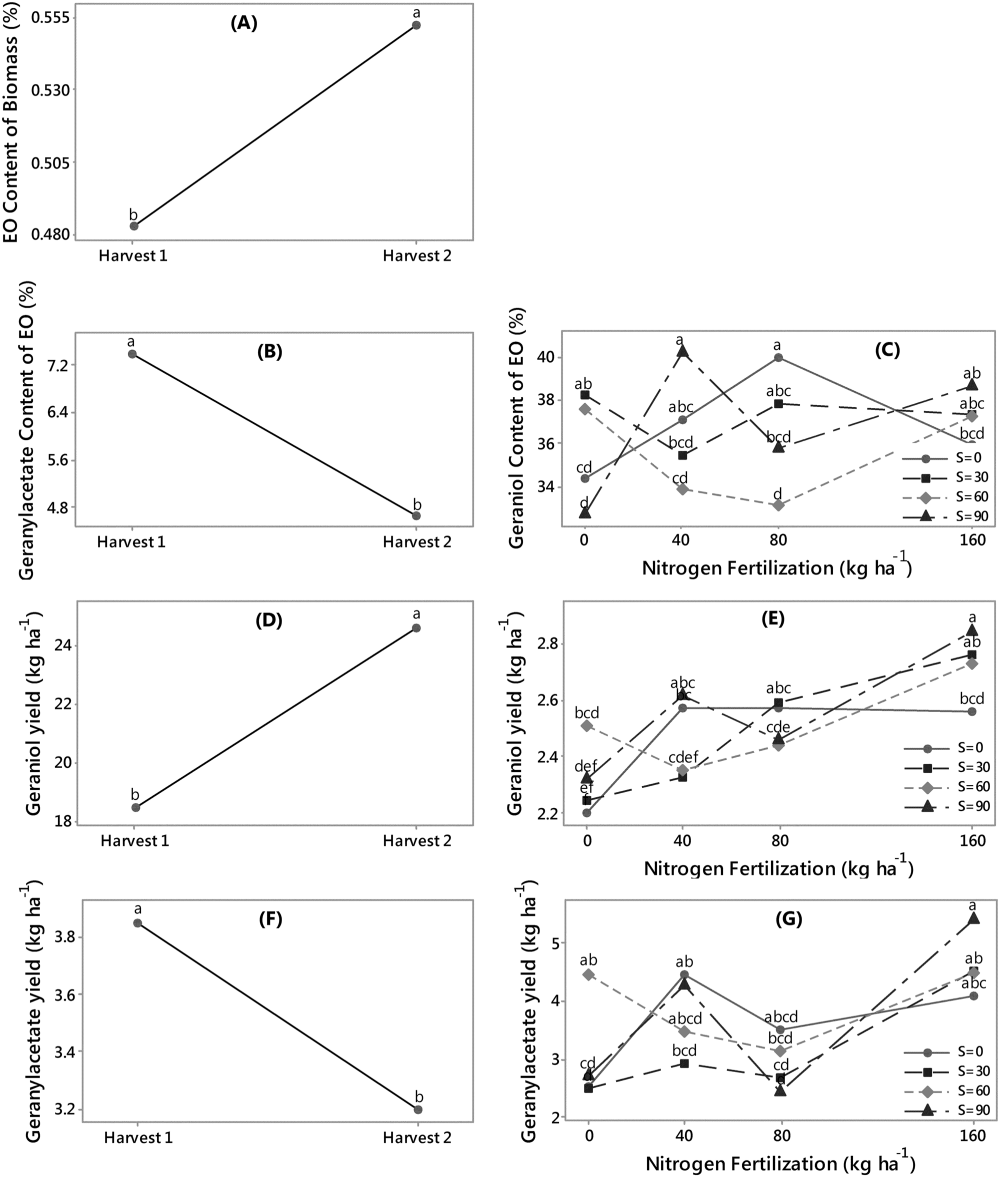
Main and interaction effect plots for palmarosa: main effect of Harvest on (A) essential oil content (%), (B) geranylacetate content (%), (D) geraniol yield, and (F) geranylacetate yield (kg ha^-1^), and interaction effect of Nitrogen and Sulfur on (C) geraniol content (%), (E) geraniol yield (kg ha^-1^), and (G) geranylacetate yield (kg ha^-1^). For each plot means sharing the same letter are not significantly different.

The yield of (*E*)-citral was higher in the N80 and N160 (32.5 and 36.2 kg ha^-1^ respectively), lower in N40, and lowest in N0 (20.4 kg ha^-1^) rate (Fig 3D). The concentration of (*E*)-citral was higher in S30 and S90 and lower in the S0 and S60 rates, indicating S30 as a possible optimum rate for maximizing the yield and the concentration of some oil constituents (Fig 2E). The yields of (*E*)-citral was higher from harvest 2 than from harvest 1 (Fig 2F).

The yields of (*Z*)-citral was highest in N160 (28.4 kg ha^-1^), lower in N80, a step lower in N40 and the lowest in N0 (15.6 kg ha^-1^) application rate (Fig 2G). The yields of (*Z*)-citral was higher from harvest 2 than from harvest 1 (Fig 2H).

The yield of *β*-caryophyllene was higher at N80 (1.18 kg ha^-1^) and lower in N0 and N40 (0.68 and 0.81 kg ha^-1^ respectively) rates (Fig 2I). Increased sulfur reduced β-caryophyllene yield in both harvest years (Fig 2J). The three-way interaction of N, S, and Harvest affected the concentrations of *β*-caryophyllene and (*Z*)-citral in the lemongrass essential oil (S1 Fig). For example, with harvest 1 and N160 rate, the addition of S increased the concentration of β-caryophyllene relative to the S0 rate. However, within harvest 2 and N80 rate, S application at 90 kg ha^-1^ had a lower concentration of β-caryophyllene relative to no application of S. Interestingly, increased N or S fertilization application had lower mean essential oil content (%) of lemongrass biomass, but increased N and S fertilization application had higher mean yield (kg ha^-1^) of most essential oil components (Fig 2D, 2E, 2G, 2I and 2J). This could have resulted from increased biomass yield (dry kg ha^-1^) from fertilization with only a slight reduction in essential oil content of the biomass. Essential oil composition varied in reports from India with 78–95% citral in lemongrass oil [25]. Some lemongrass clones are rich in (*E*)-citral (66–73%), with approximately 10% (*Z*)-citral, and 8–10% linalool [26]. N application rates, other agroecological conditions, and even leaf position within one plant were shown to alter essential oil composition of lemongrass [27]. Additionally, as the response of β-caryophyllene and (*Z*)-citral concentrations in our study did not have a direct trend in relation to fertilization rates we may assume the presence of other factors modifying the concentrations of these two terpenes in lemongrass oil.

### *C*. *martinii* essential oil responses

Harvest had a significant effect on essential oil content of palmarosa biomass which was higher in harvest 2 than harvest 1 (Fig 3A). Harvest also had a significant effect on geranylacetone content of palmarosa essential oil (Fig 3B). Interaction between N and S had significant effect on the concentration of geraniol in palmarosa essential oil (Fig 3C). Geranial yield was positively affected by harvest (Fig 3D) and nitrogen and sulfur interactions (Fig 3E). Geranylacetate yield was negatively affected by harvest (Fig 3F) and nitrogen and sulfur interaction (Fig 3G). Geranial concentration in palmarosa oil ranged between 70–85% and geranylacetate varied from 4 to 15% [28]. Other studies reported geraniol at 82% and geranylacetate of 10% [29], or up to 93% geranial, 3–4% linalool, and 2% geranylacetate [30].

### Biomass composition and recalcitrance

The composition of lemongrass and palmarosa biomass was investigated to determine feasibility of use in lignocellulosic biofuel and high-value coproducts fermentation. Lemongrass and palmarosa oil composition can be altered by many factors; in this work we investigate the impact of nitrogen and sulfur fertilization rate, and plant age (harvest year). The general biosynthetic pathways of lemongrass and palmarosa oil were recently reviewed by [31]. Lignin has been identified as a major inhibitor of lignocellulosic biomass fermentation. Specifically, recalcitrance of lignocellulosic biomass to enzyme degradation and fermentation has been linked specifically to the overall S/G ratio of lignin subunits in biomass [13]. Palmarosa biomass had the largest lignin fraction and the highest S/G ratio (Table 1). Stem tissues of both lemongrass and palmarosa biomass had greater lignin fraction and a higher S/G ratio than leaf tissue. Average glucose yield from lemongrass whole biomass was about double that of palmarosa whole biomass (Table 1). However, palmarosa whole biomass had 22.2% more cellulose content than lemongrass whole biomass. This suggests that palmarosa biomass should produce more ethanol than lemongrass if complete hydrolysis is achieved and no inhibition results from hydrolysis products or native metabolites. Xylan content was similar for both lemongrass and palmarosa biomass (Table 1). Xylans have previously been found to inhibit biomass hydrolysis by endoglucanases and cellobiohydrases [32], however the different ethanol yields between lemongrass and palmarosa do not seem to be due to xylan content.

**Table 1:** Lemongrass and palmarosa biomass without pretreatment fraction composition and enzymatic saccharification efficiency.

Biomass Type	Biomass^a^	Fraction	Lignin (%)	S/G ratio	Cellulose content (g g^-1^ biomass)	Cellulose release (g g^-1^ biomass)	Average Cellulose Yield^b^ (%)	Xylan content (g g^-1^ biomass)	Xylan release (g g^-1^ biomass)	Average Xylan Yield^b^ (%)
**Lemongrass**	**NE**	Whole	19.87	0.48	0.2827	0.1629	51.86	0.2006	0.1411	61.88
		Leaf	18.77	0.46	0.2713	0.1768	58.67	0.1848	0.1506	71.70
		Stem	22.23	0.54	0.3486	0.1863	48.10	0.2002	0.1404	61.68
**Lemongrass**	**EX**	Whole	21.39	0.46	0.3024	0.1706	50.77	0.2080	0.1483	62.72
		Leaf	20.30	0.44	0.2463	0.1663	60.79	0.1862	0.1472	69.56
		Stem	24.20	0.56	0.3861	0.1758	40.97	0.3861	0.1511	64.19
**Palmarosa**	**NE**	Whole	23.76	0.65	0.3676	0.1165	28.53	0.1967	0.1298	58.06
		Leaf	16.91	0.42	0.2276	0.1872	74.03	0.1682	0.1218	63.75
		Stem	27.57	0.77	0.4158	0.0850	18.40	0.1985	0.1289	57.13
**Palmarosa**	**EX**	Whole	24.06	0.68	0.3885	0.1174	27.19	0.2052	0.1339	57.42
		Leaf	18.79	0.48	0.2714	0.1710	56.71	0.1749	0.1281	64.49
		Stem	28.79	0.84	0.3804	0.0950	22.49	0.2074	0.1380	58.57

^a^ NE—not-extracted; EX—extracted biomass.

^b^ Average Yield = Release (g g^-1^ cellulose or xylan)/Content (g g^-1^ biomass).

**Fig 4:**
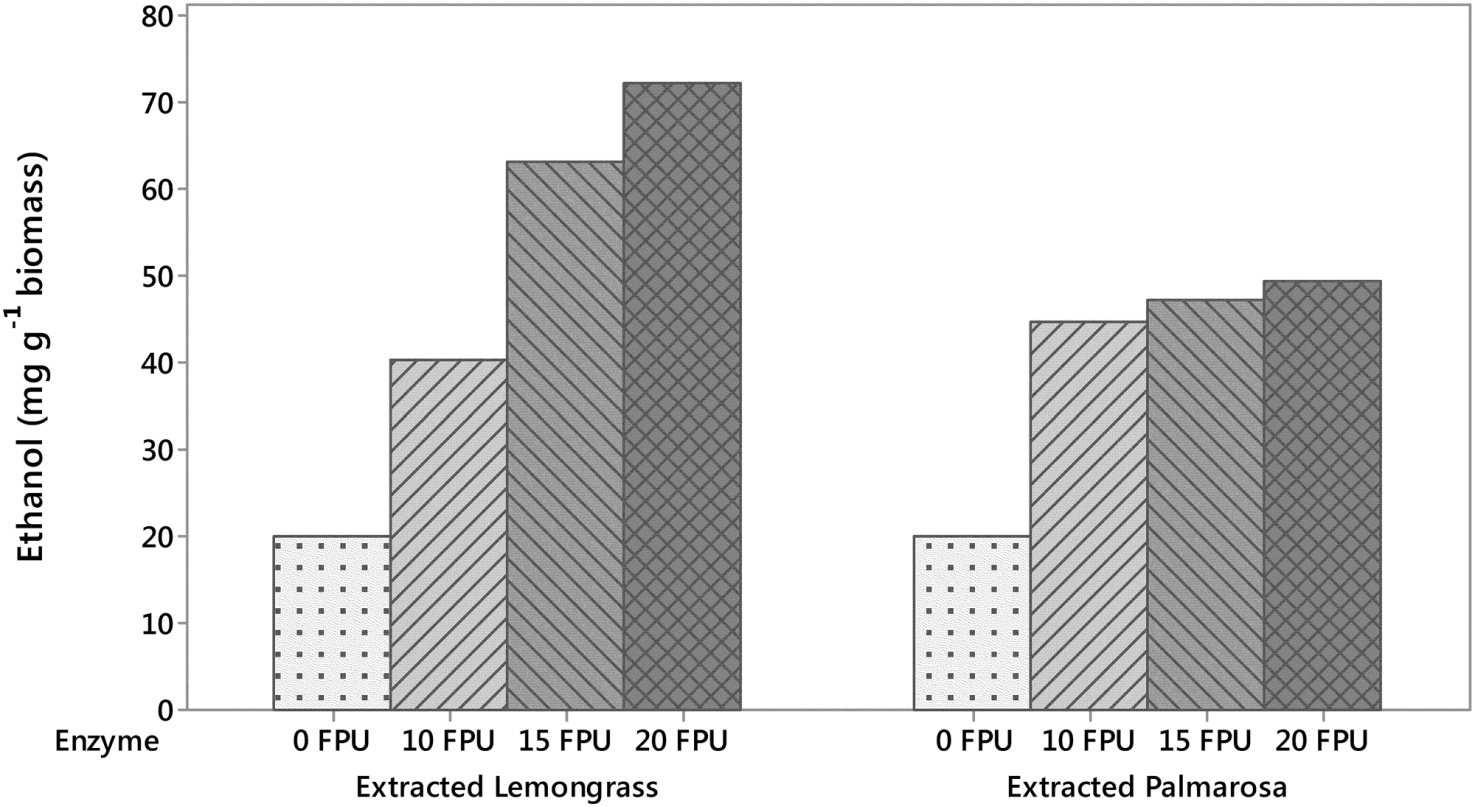
Bench top simultaneous saccharification and fermentation (SSF) ethanol yield from extracted whole biomass based on filter paper units of enzymes. Fermentation of lemongrass biomass reached maximum yields at enzyme concentrations of 15 FPU g^-1^ biomass. Fermentation of palmarosa biomass reached maximum yields at an enzyme concentration of 10 FPU g^-1^ biomass.

Palmarosa recalcitrance to enzymatic degradation was mirrored in principal during SSF optimization. Lemongrass biomass showed a coordinated response to enzyme dosage with a plateau between 15 (filter paper units, FPU g^-1^ biomass) and 20 (FPU g^-1^ biomass), whereas palmarosa biomass yielded a maximum between 10 (FPU g^-1^ biomass) and 15 (FPU g^-1^ biomass) (Fig 4). Steam distillation of biomass to extract essential oils did not affect lignin fraction, lignin S/G ratio, enzymatic saccharification cellulose release, or enzymatic saccharification xylose release (Table 1). These results suggest that the steam distillation does not pretreat biomass.

### Ethanol fermentation and inhibition from biomass metabolites

**Fig 5:**
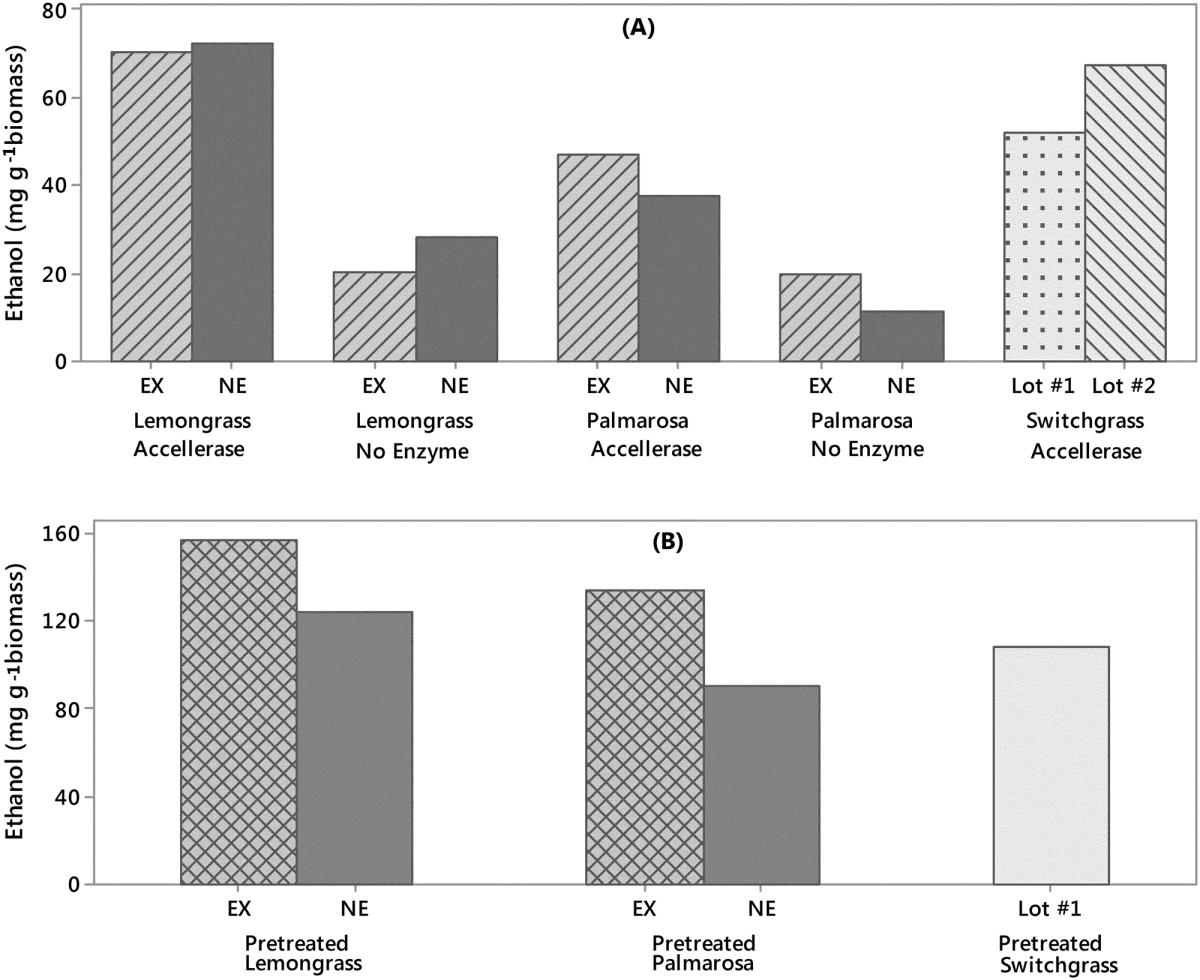
Final ethanol yield (mg g^-1^ biomass) of lemongrass and palmarosa biomass that was (EX) or was not (NE) previously extracted for essential oils in comparison to two lots of BioEnergy Science Center (BESC) control switchgrass. A) Final ethanol concentration of biomass that was not pretreated in fermentation liquids. B) Final ethanol yield (mg g^-1^ biomass) of dilute acid pretreated lemongrass and palmarosa biomass that was (EX) or was not (NE) extracted for essential oils in comparison to lot #1 BESC control switchgrass.

Benchtop-scale simultaneous saccharification and fermentation (SSF) was carried out to investigate the potential to produce biofuels, i.e. ethanol in this work, from lemongrass and palmarosa biomass and how the role of secondary metabolites found in these two species might affect biofuel production efficiency. Bench-top SSF experiments model lignocellulosic biorefinery process to determine suitability of biomass after pretreatment. Lemongrass extracted (EX) biomass yielded less ethanol than not-extracted biomass (NE), but EX palmarosa biomass yielded more ethanol than NE palmarosa biomass (Fig 5A). These patterns were the same in biomass treated with and without enzyme, suggesting that results were from biomass properties, rather than inhibition of enzymatic hydrolysis. Lemongrass and palmarosa essential oils were found to interact with *S*. *cerevisiae* cell membranes and cause the leakage of ions until cellular death [6,7]. These two reports found a concentration of 0.1% for either lemongrass or palmarosa essential oil was toxic to *S*. *cerevisiae*. The major essential oil constituents of lemongrass (citral) and palmarosa (geraniol) have low solubility in water because they are primarily nonpolar terpenoid hydrocarbons; however, geranial is 38.8% more soluble in water, 420 mg L^-1^ and 686 mg L^-1^ at 20°C respectively (GESTIS database, October 2012). Therefore, fermentation of palmarosa biomass could result in *S*. *cerevisiae* toxicity in relatively lower biomass concentrations than lemongrass or switchgrass as observed (Fig 5A). These considerations support two different mechanisms for the difference in ethanol fermentation potential from lemongrass and palmarosa. First, lemongrass biomass losses fermentable glucose by steam distillation which leads to higher ethanol yields from not-extracted biomass. Alternatively, the essential oil present in palmarosa may inhibit *S*. *cerevisiae* fermentation, which leads to higher ethanol yields from biomass that has had the essential oils removed through steam distillation.

Overall, both extracted and not-extracted lemongrass biomass yielded similar amounts of ethanol as BESC switchgrass and palmarosa biomass yielded the least amount of ethanol of all the biomass types tested. The difference in ethanol yield between the two lots of BESC switchgrass can be explained further by the remaining biomass fractions in the fermentation liquid. Lot #1 had three times the amount of residual cellulose (glucose) after fermentation compared to lot #2 (S2 Table). BESC switchgrass lot #2 had a similar amount of cellulose leftover after fermentation as lemongrass and palmarosa biomass.

Pretreatment of biomass universally increased production of ethanol from biomass (Fig 5B; S2 Table). It was unclear whether pretreatment of biomass would result in increased concentrations of inhibitory secondary metabolites. After pretreatment, extracted lemongrass and palmarosa biomass produce more ethanol than biomass that had not been extracted (Fig 5B). Interestingly, palmarosa biomass turned a dull red after dilute acid pretreatment whereas all other biomass samples remained brown (data not shown). Only NE palmarosa biomass had remaining glucose in the fermentation liquid of all pretreated biomass (S2 Table). This supports the previous observation that palmarosa biomass will yield toxic concentrations of essential oils at lower biomass concentrations. In short, pretreatment may break open more cells which would provide better enzyme access in palmarosa biomass resulting in increased concentrations of essential oils in fermentation liquids. In future experiments, analysis of fermentation liquids for essential oil metabolites would yield interesting observations and help to predict specific concentrations of coproducts that are inhibitory to biofuel production in larger processes.

### From field to fermenter: estimated market values of coproduction

Finally, it is important to consider the potential of high-value, low-volume coproducts to offset the inherent low-value, high-volume economics of biofuel production. Coupling our biomass and essential oil agronomic data and the SSF ethanol production data the market value of lemongrass and palmarosa biomass can be estimated. The second harvest year had a mean biomass yield of 128 000 kg ha^-1^ for lemongrass and 151 000 kg ha^-1^ for palmarosa. Pretreated lemongrass yield of 156 (mg g^-1^ biomass) or a volume of 198 (mL g^-1^ biomass) considering ethanol’s density of 0.789 g mL^-1^ (Fig 5B). Pretreated palmarosa had an ethanol yield of 134 (mg g^-1^ biomass) or 170 (mL g^-1^ biomass)^-1^. This equates to theoretical yields of roughly 2541 L ethanol ha^-1^ for lemongrass and 2569 L ethanol ha^-1^ for palmarosa biomass, or $1600 and $1620, respectively, for current spot ethanol prices at $0.63 L^-1^ (Chicago Board of Trade October 2012). The ethanol production for these dual-use crops falls within the theoretical maximum ethanol production (100% conversion assumed) range, 1749 L ha^-1^ to 3691 L ha^-1^, for switchgrass [33] and compares to actual observed yield means from switchgrass, 3091 L ha^-1^ [34]. A total of $1950 ha^-1^ can be produced from switchgrass fields, assuming 3091 L ha^-1^ for average switchgrass production as in [34].

Previous reports in India note a value of $10.00 (USD) for lemongrass essential oil per kilogram and $15.00 for palmarosa essential oil [25,30]. It is likely that these essential oils would have a greater value in the United States or in international markets [18]. The mean value for the second-year harvest for lemongrass essential oil yield was 85.7 kg ha^-1^ and 67 kg ha^-1^ for palmarosa. Therefore, lemongrass would yield $857 ha^-1^ and palmarosa would yield $1005 ha^-1^ in essential oil coproduct sales. At a refinery, this translates to an additional $66.80 Mg^-1^ for lemongrass and $66.51 Mg^-1^ for palmarosa based on the mean biomass production values per hectare. Assuming a spot ethanol prices of $0.63 L^-1^, a total of $2457 lemongrass ha^-1^ and $2625 palmarosa ha^-1^ for ethanol and essential oil production can be produced while only $1950 for switchgrass spot ethanol ha^-1^ alone can be realized currently. While these results help to quantify the value of coproducts to the biofuels industry, these estimates are derived from small plots and bench-top ethanol fermentation scales. Plot size was previously found to not significantly affect biomass yield from test plots [21], and so it is likely that the size of test plots will affect the economic estimates. However, further investigation across multiple years and growing climates will be needed to determine whether lemongrass and palmarosa biomass could be used as feasible dual-use lignocellulosic feedstocks.

## Conclusions

We report agronomic production, essential oil, and ethanol production from two novel dual-use lignocellulosic crops. Extrapolation of the results lead to an ethanol yield of 2541 L ha^**-1**^ of lemongrass and 2569 L ha^-1^ of palmarosa biomass with an additional essential oil yield of 85.7 kg ha^**-1**^ and 67 kg ha^**-1**^. This leads to a combined value of $2457 ha^-1^ lemongrass and $2625 ha^-1^ palmarosa for ethanol and essential oil compared to $1950 for switchgrass spot ethanol ha^-1^ alone. These results support the potential value of coproduct economics in the emerging biofuel industry, and have identified two feasible dual-uses for biofuel and coproduct commercialization.

## Supporting Information

S1 Fig Interaction effect of nitrogen×sulfur×harvest on β-caryophyllene content of essential oil (%) [(A) Harvest 1, and (B) Harvest 2] and (*Z*)-citral content of essential oil (%) [(C) Harvest 1, and (D) Harvest 2] in lemongrass essential oil. (TIF)

S1 Table P-values showing the effect of N, S and harvest on essential oil (EO) content (%), and the composition and yield of *β*-caryophyllene, (*Z*)-citral and (*E*)-citral for Lemongrass; and on EO content (%), and the composition and yield of geraniol and geranylacetate for Palmarosa. (DOCX)

S2 Table Biomass fractions remaining in fermentation liquid after SSF for enzyme optimization, dried harvested biomass, and pretreated biomass as determined by HPLC. (DOCX)
