# Supplementary material for: Ethanol and High-Value Terpene Co-Production from Lignocellulosic Biomass of Cymbopogon flexuosus and Cymbopogon martinii
Source: PLoS One. 2015 Oct 5;10(10):e0139195. doi: 10.1371/journal.pone.0139195 (PMC4593581; doi:10.1371/journal.pone.0139195)
Supplement: S1 Table — (DOCX) [file pone.0139195.s002.docx]

**S1 Table.** P-values showing the effect of N, S and harvest on essential oil (EO) content (%), and the composition and yield of *β*-caryophyllene, (*Z*)-citral and (*E*)-citral for Lemongrass; and on EO content (%), and the composition and yield of geraniol and geranylacetate for Palmarosa.

| Source |  | Lemongrass | | | | | | |  | Palmarosa | | | | | |
| --- | --- | --- | --- | --- | --- | --- | --- | --- | --- | --- | --- | --- | --- | --- | --- |
|  | EO content  of biomass | Content in EO (%) | | |  | Yield (kg ha^-1^) | | |  | EO content of biomass | Content in EO (%) | |  | Yield (kg ha^-1^) | |
|  |  | β-caryophyllene | (Z)-citral | (E)-citral |  | β-caryophyllene yield | (Z)- citral  yield | (E)- citral  yield |  |  | Geraniol | Geranyl acetate |  | Geraniol yield | Geranyl acetate yield |
| Block | 0.128 | 0.069 | 0.270 | 0.136 |  | 0.071 | 0.811 | 0.661 |  | 0.006 | 0.467 | 0.001 |  | 0.001 | 0.001 |
| N | **0.029**^1^ | 0.009 | 0.832 | 0.262 |  | **0.001** | **0.001** | **0.001** |  | 0.598 | 0.474 | **0.001** |  | 0.001 | 0.001 |
| S | **0.024** | 0.688 | 0.025 | **0.001** |  | 0.153 | 0.704 | 0.262 |  | 0.958 | 0.318 | 0.274 |  | 0.394 | 0.142 |
| N×S | 0.316 | 0.032 | 0.649 | 0.458 |  | 0.157 | 0.514 | 0.281 |  | 0.927 | **0.001** | 0.451 |  | **0.003** | **0.034** |
| Harvest | **0.002** | 0.001 | 0.964 | 0.367 |  | 0.007 | **0.046** | **0.047** |  | **0.001** | 0.705 | **0.001** |  | **0.001** | **0.028** |
| N×Harvest | 0.587 | 0.432 | 0.382 | **0.086** |  | 0.506 | 0.832 | 0.670 |  | 0.444 | 0.962 | 0.701 |  | 0.718 | 0.574 |
| S×Harvest | 0.648 | 0.003 | 0.390 | 0.703 |  | **0.013** | 0.245 | 0.236 |  | 0.390 | 0.526 | 0.970 |  | 0.753 | 0.953 |
| N×S×Harvest | 0.944 | **0.089** | **0.086** | 0.236 |  | 0.416 | 0.277 | 0.127 |  | 0.831 | 0.974 | 0.444 |  | 0.842 | 0.451 |
| ^1^Significant effects that require multiple means comparison are shown in bold. | | | | | | | | | | | | | | | |
