# Supplementary material for: Ethanol and High-Value Terpene Co-Production from Lignocellulosic Biomass of Cymbopogon flexuosus and Cymbopogon martinii
Source: PLoS One. 2015 Oct 5;10(10):e0139195. doi: 10.1371/journal.pone.0139195 (PMC4593581; doi:10.1371/journal.pone.0139195)
Supplement: S2 Table — (DOCX) [file pone.0139195.s003.docx]

S2 Table. Biomass fractions remaining in fermentation liquid after SSF for enzyme optimization, dried harvested biomass, and pretreated biomass as determined by HPLC.

| Enzyme Optimization | Enzyme Concentration  (FPU g^-1^ biomass) | Biomass Fraction Remaining in Fermentation Liquid (mg g^-1^ biomass) | | | | | |
| --- | --- | --- | --- | --- | --- | --- | --- |
|  |  | Cellobiose | Glucose | Xylose | Arabinose | Acetic acid | Ethanol |
| Lemongrass Extracted | 0 | 3.71 | 0.97 | 0.00 | 1.58 | 2.16 | 20.05 |
|  | 10 | 1.85 | 1.75 | 10.16 | 3.30 | 4.59 | 40.46 |
|  | 15 | 3.46 | 0.00 | 16.06 | 4.76 | 6.34 | 63.16 |
|  | 20 | 3.01 | 1.83 | 13.10 | 5.54 | 7.33 | 72.24 |
| Palmarosa Extracted | 0 | 1.44 | 0.00 | 0.00 | 1.23 | 1.85 | 20.10 |
|  | 10 | 2.04 | 2.02 | 11.17 | 3.64 | 4.83 | 44.79 |
|  | 15 | 1.13 | 2.17 | 20.89 | 4.77 | 4.66 | 47.16 |
|  | 20 | 2.12 | 2.14 | 13.73 | 4.26 | 6.27 | 49.54 |
| No Pretreatment Biomass Fermentation | Enzyme Concentration  (FPU g^-1^ biomass) | Cellobiose | Glucose | Xylose | Arabinose | Acetic acid | Ethanol |
| Lemongrass Extracted | 15 | 3.04 | 0.00 | 13.48 | 6.02 | 7.39 | 70.38 |
|  | 0 | 3.77 | 1.13 | 0.00 | 1.91 | 2.13 | 20.30 |
| Lemongrass Not-extracted | 15 | 3.57 | 1.50 | 14.99 | 5.70 | 7.00 | 72.45 |
|  | 0 | 5.08 | 1.27 | 9.25 | 1.88 | 3.25 | 28.30 |
| Palmarosa Extracted | 15 | 2.07 | 1.82 | 14.97 | 4.80 | 5.66 | 47.38 |
|  | 0 | 3.52 | 2.07 | 0.00 | 1.57 | 2.13 | 19.89 |
| Palmarosa Not-extracted | 15 | 2.43 | 1.31 | 15.57 | 4.63 | 4.66 | 37.72 |
|  | 0 | 3.59 | 1.60 | 0.00 | 1.26 | 1.77 | 11.48 |
| Switchgrass Lot #1 | 15 | 1.21 | 3.22 | 13.87 | 3.34 | 6.82 | 52.11 |
| No Biomass | 15 | 0.31 | 0.00 | 0.46 | 0.00 | 0.00 | 0.00 |
| Switchgrass Lot #2 | 15 | 1.89 | 1.11 | 11.29 | 3.76 | 5.91 | 67.64 |
| Pretreated Biomass | Enzyme Concentration  (FPU g^-1^ biomass) | Cellobiose | Glucose | Xylose | Arabinose | Acetic acid | Ethanol |
| Lemongrass Extracted | 15 | 1.61 | 0.00 | 12.88 | 0.83 | 14.11 | 156.27 |
| Lemongrass Not-extracted | 15 | 1.79 | 0.00 | 18.55 | 1.55 | 14.29 | 124.21 |
| Palmarosa Extracted | 15 | 1.36 | 0.00 | 13.31 | 0.85 | 9.99 | 134.15 |
| Palmarosa Not-extracted | 15 | 0.85 | 1.77 | 13.22 | 0.68 | 6.39 | 90.11 |
| Switchgrass Lot #1 | 15 | 2.39 | 0.00 | 21.42 | 1.03 | 12.36 | 108.23 |
| No Biomass | 15 | 0.76 | 0.00 | 1.39 | 0.00 | 0.00 | 0.00 |
